# Degos disease complicated by constrictive pericarditis in remote phase: a case report

**DOI:** 10.1186/s13019-022-01810-0

**Published:** 2022-04-01

**Authors:** Yuki Tadokoro, Tadashi Kitamura, Tetsuya Horai, Kagami Miyaji

**Affiliations:** Department of Cardiovascular Surgery, Kitasato University School of Medicine, 1-15-1 Kitasato, Minami, Sagamihara, Kanagawa 252-0374 Japan

**Keywords:** Degos disease, Constrictive pericarditis, Heart failure

## Abstract

**Background:**

Degos disease, also known as malignant atrophic papulosis, is characterised by cutaneous manifestations due to chronic thrombo-obliterative vasculopathy. There have been reports of the rare late-onset Degos disease complicated by constrictive pericarditis (CP). This study reports a case of CP caused by Degos disease that developed 20 years after diagnosis.

**Case presentation:**

A 62-year-old woman who had been taking aspirin for 20 years for Degos disease was hospitalised for worsening of heart failure. The patient was diagnosed with CP and underwent pericardiectomy. Pathological findings suggested the involvement of Degos disease. The postoperative course was uneventful, and her heart failure and Degos disease did not worsen.

**Conclusions:**

The study findings suggests that Degos disease can cause long-term CP. Aspirin effectively inhibited the progression of Degos disease, and surgical treatment was necessary when heart failure due to CP was refractory to treatment.

## Background

Degos disease, also known as malignant atrophic papulosis, is rare. To date, approximately 200 cases have been reported in the literature. Degos disease is characterised by cutaneous signs, such as central porcelain-white atrophic papules with an erythematous telangiectatic rim caused by chronic thrombo-obliterative vasculopathy [1, 2]. However, there have been only few reports of constrictive pericarditis (CP) caused by Degos disease [3, 4]. To the best of our knowledge, this is the first report of CP caused by Degos disease that developed 20 years after diagnosis. This study presents our fidings of surgical intervention performed for CP caused by Degos disease in a patient who presented with treatment-refractory heart failure.

## Case presentation

A 67-year-old woman was admitted to our cardiology department for dyspnoea. Her medical history included hypertension, atrial fibrillation, diabetes mellitus, and Degos disease, and she had been taking low-dose aspirin for 20 years.

At the time of diagnosis, she exhibited cutaneous signs, and her histopathological examination revealed perivascular lymphocytic infiltration with distinct mucin deposition. These lesions were associated with Degos disease [1, 2]. No systemic symptoms were observed. Three years ago, gastrointestinal endoscopy revealed a small intestinal lesion, which was suspected to be a systemic manifestation of Degos disease [5].

**Fig. 1:**
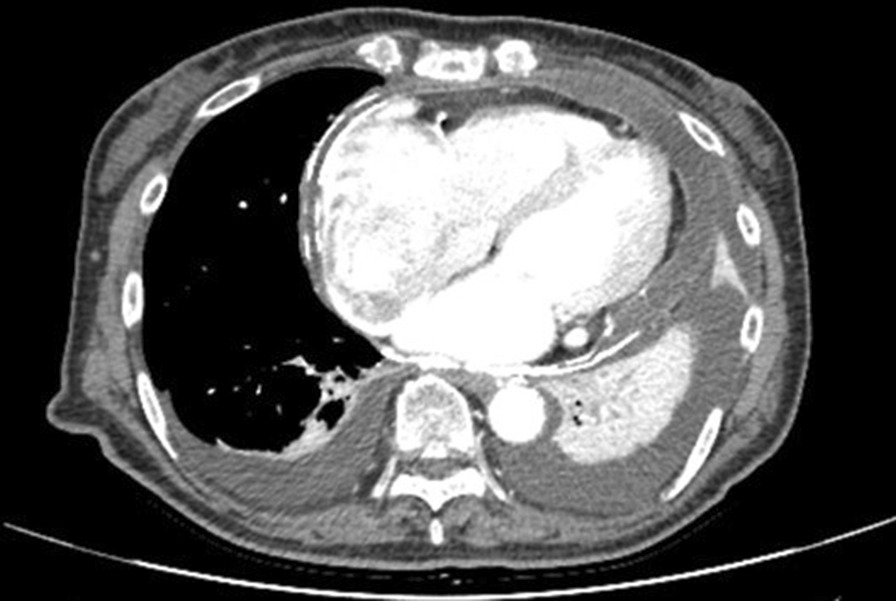
Computed tomography demonstrating bilateral pleural effusion and pericardial effusion with marked calcification of the pericardium

On admission, her blood pressure was 110/62 mmHg, and her heart rate was 99 beats/min with atrial fibrillation. Physical examination revealed liver enlargement, jugular vein distension with Kussmaul’s sign, and limb oedema. Chest radiography revealed bilateral pleural effusion and calcification of the pericardium. Bilateral pleural effusion and pericardial effusion with marked calcification of the pericardium were noted on computed tomography (Fig. 1).

**Fig. 2:**
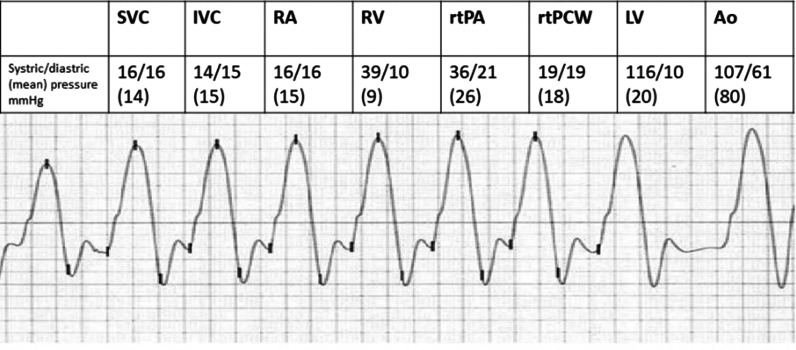
Cardiac catheterisation demonstrating equal right and left ventricular end-diastolic pressures and square root signs. SVC: superior vena cava; IVC: inferior vena cava; RA: right atrium; RV: right ventricle; rtPA: right pulmonary artery; rtPCW: right pulmonary capillary wedge pressure; LV: left ventricle; Ao: aorta

Cardiac catheterisation revealed equal right and left ventricular end-diastolic pressures and square root signs (Fig. 2). No coronary artery stenosis was observed. Echocardiography revealed pericardial thickening, pericardial effusion, ventricular septal paradoxical motion, septal bounce, and a normal left ventricular ejection fraction. The cutaneous signs were similar to those observed 20 years ago. Endoscopy revealed the same findings 3 years ago [5].

Despite optimal medical treatment, her heart failure did not improve, and the patient became catecholamine dependent. Therefore, surgical pericardiectomy was performed.

**Fig. 3:**
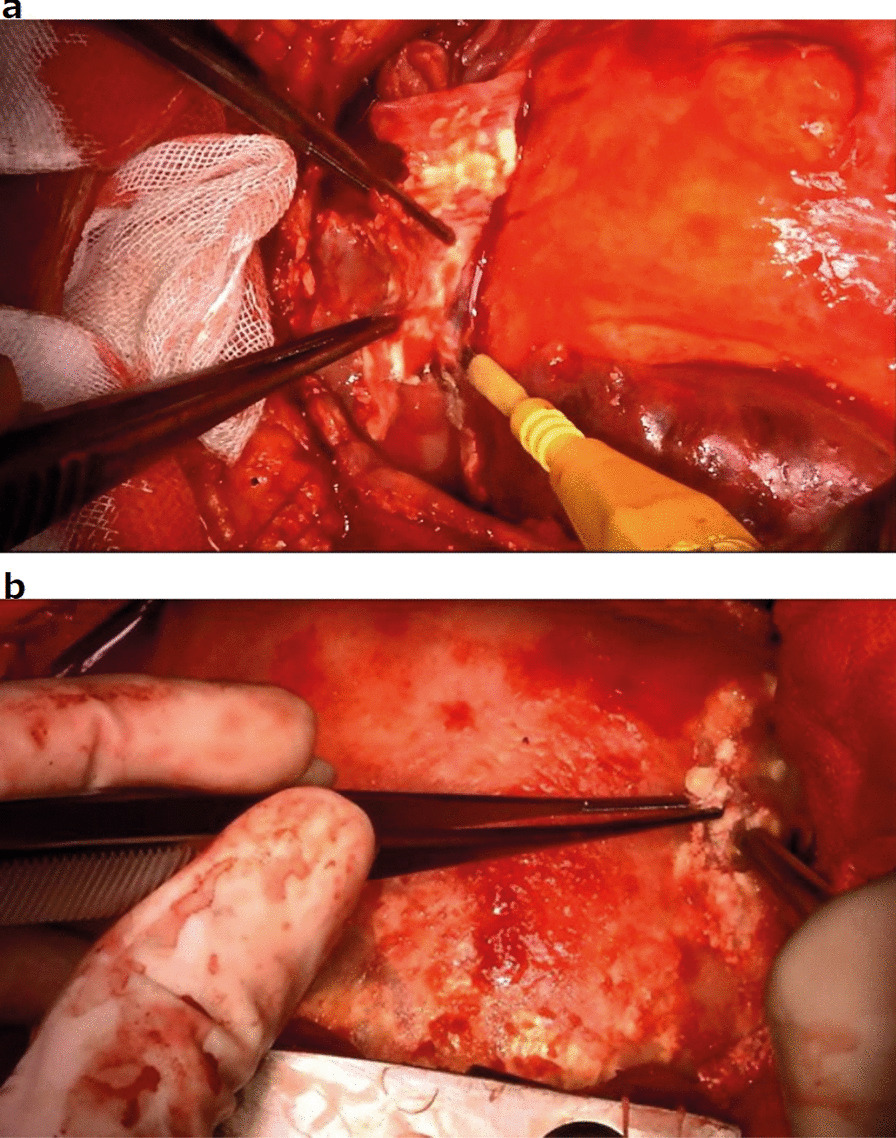
Intraoperative gross findings. **a** The inside of the pericardial sac displays adhesions with some calcification; **b** A high degree of calcification in the myocardium is observed

**Fig. 4:**
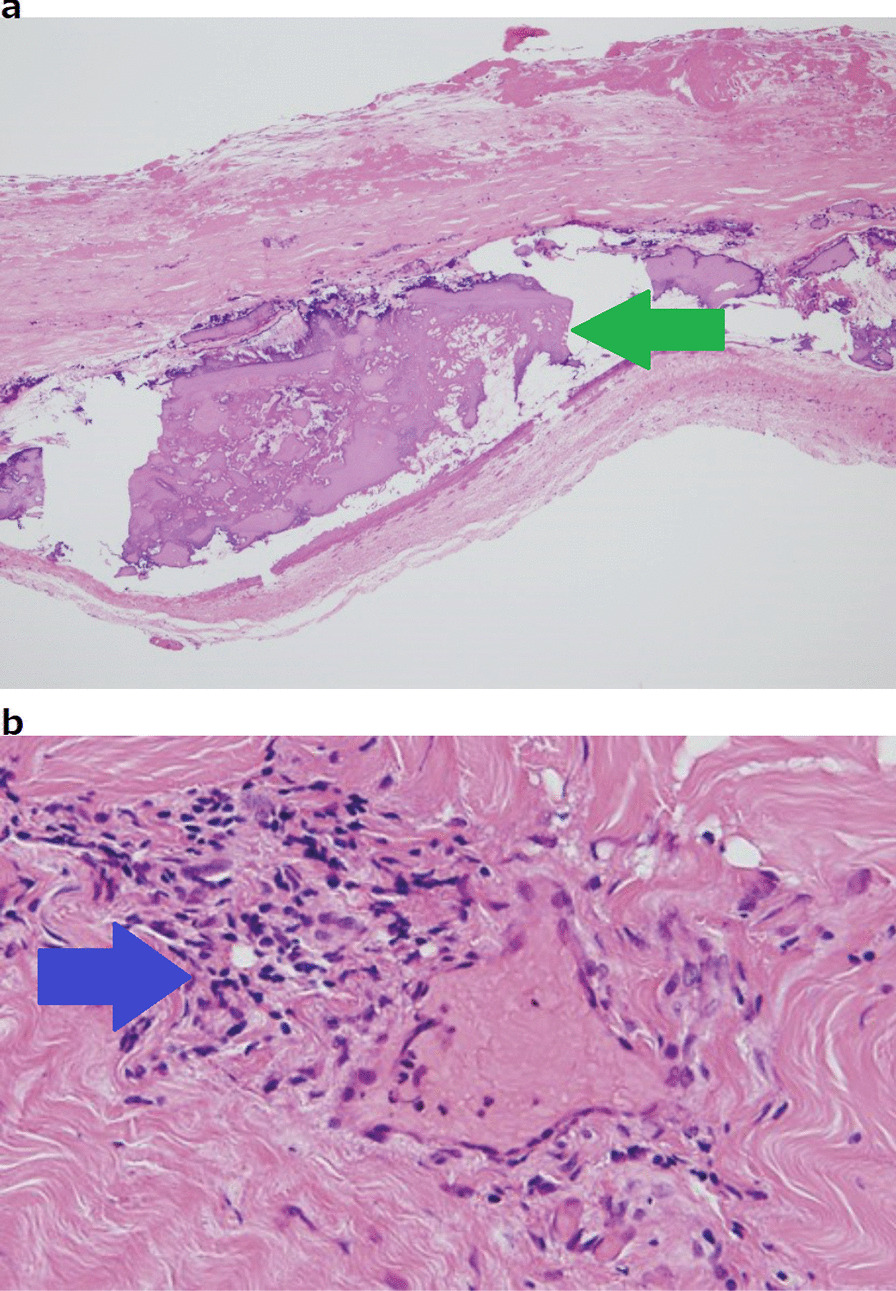
Histopathological findings. **a** There is a high degree of calcification and fibrosis in the pericardium (green arrow); **b** Lymphocytic infiltration around the pericardial vessels (blue arrow)

During the operation, the pericardium was markedly thickened and calcified. The pericardium was incised, and 200 ml of bloody fluid was suctioned. Inside the pericardial sac, there were adhesions with some calcification (Fig. 3a) that partly infiltrated the myocardium (Fig. 3b). The thickened pericardium was then thoroughly resected. The central venous pressure decreased from 30 to 16 mm Hg, and the cardiac diastolic capacity improved. Histopathological examination of the pericardium revealed a high degree of fibrosis, vitrification, and calcification of the pericardium. Lymphocytic infiltration was observed around the pericardial vessels (Fig. 4a, b). The postoperative course was uneventful. The patient was extubated on day 1, discharged from the intensive care unit on day 2, and discharged from the hospital on day 18. After surgery, the patient received aspirin, furosemide, spironolactone, bisoprolol, and perindopril erbumine treatment for 4 years. Her heart failure did not worsen.

## Discussion and conclusions

Pierce et al. had reported a case of chronic pleuritis and pericarditis in a 32-year-old woman with Degos disease [3], wherein the patient developed heart failure and required surgical treatment. In this previous study, histopathological examination revealed a calcified and fibrotic epicardium, similar to that noted in our case, but there was no proliferative vasculitis of Degos disease.

Few studies have reported cases of CP due to Degos disease. Of these reported cases, surgery was performed in only two cases [3, 6]. In these reports, histopathological examination did not show any vasculitis or any other findings specific to Degos disease, other than general findings such as calcification of the pericardium. In our case, lymphocytic infiltration was present around the pericardial vessels, which is suggestive of Degos disease.

According to Theodoridis et al., systemic signs were present in 29% of patients with Degos disease. A previous study has reported that organ involvement began within the first 7 years of disease, and the mean survival time from the development of systemic disease was 0.9 years [2]. However, our patient who took aspirin did not develop a systemic disease until 17 years after diagnosis. To the best of our knowledge, the time to onset of CP after 20 years after diagnosis is the longest reported. Since the current study is a case report, a general conclusion cannot be made. However, Yukiiri et al. Have reported CP caused by untreated Degos disease and medically treated with aspirin, dipyridamole, and furosemide [4].

Therefore, aspirin was found to effectively inhibit the progression of Degos disease. In summary, Degos disease can cause long-term CP. Aspirin effectively inhibits the progression of Degos disease, and surgical treatment is necessary when heart failure due to CP is refractory to treatment.

## Data Availability

Not applicable.

## Abbreviation

CP: Constrictive pericarditis
